# Transcriptomic analysis of *Saccharomyces cerevisiae* x *Saccharomyces* *kudriavzevii* hybrids during low temperature winemaking

**DOI:** 10.12688/f1000research.11550.3

**Published:** 2017-09-12

**Authors:** Jordi Tronchoni, Estéfani García-Ríos, Jose Manuel Guillamón, Amparo Querol, Roberto Pérez-Torrado

**Affiliations:** 1 Food Biotechnology Department, Institute of Agrochemistry and Food Technology (IATA-CSIC), Paterna, Valencia, Spain; 2 Instituto de Ciencias de la Vid y del Vino (ICVV), Gobierno de La Rioja-CSIC-Universidad de La Rioja, Logroño, La Rioja, Spain

**Keywords:** Saccharomyces cerevisiae, Saccharomyces kudriavzevii, hybrids, cold stress, winemaking

## Abstract

Background: Although *Saccharomyces cerevisiae* is the most frequently isolated species in wine fermentation, and the most studied species, other species and interspecific hybrids have greatly attracted the interest of researchers in this field in the last few years, given their potential to solve new winemaking industry challenges. *S. cerevisiae* x *S. kudriavzevii* hybrids exhibit good fermentative capabilities at low temperatures, and produce wines with smaller alcohol quantities and larger glycerol quantities, which can be very useful to solve challenges in the winemaking industry such as the necessity to enhance the aroma profile.

Methods: In this study, we performed a transcriptomic study of *S. cerevisiae* x *S. kudriavzevii* hybrids in low temperature winemaking conditions.

Results: The results revealed that the hybrids have acquired both fermentative abilities and cold adaptation abilities, attributed to *S. cerevisiae* and *S. kudriavzevii* parental species, respectively, showcasing their industrially relevant characteristics. For several key genes, we also studied the contribution to gene expression of each of the alleles of *S. cerevisiae* and *S. kudriavzevii* in the *S. cerevisiae* x *S. kudriavzevii *hybrids. From the results, it is not clear how important the differential expression of the specific parental alleles is to the phenotype of the hybrids.

Conclusions: This study shows that the fermentative abilities of *S. cerevisiae* x *S. kudriavzevii *hybrids at low temperatures do not seem to result from differential expression of specific parental alleles of the key genes involved in this phenotype.

## Introduction

Wine yeasts are specialised organisms adapted to restrictive environmental conditions created by human technology. The most frequently isolated species in wine fermentations is *Saccharomyces cerevisiae,* but other species of the *Saccharomyces* genus and their interspecific hybrids are also present in the final wine fermentations. These species have attracted significant interest in the last years due to their potential in solving the main challenges the winemaking industry faces, such as the enhancement of aroma. There is a trend in winemaking to decrease fermentation temperatures, which improves the wine aromatic profile. The wine industry has yeasts that are commercialized as cryotolerant *S. cerevisiae* yeasts, however most of them do not show desirable fermentation performance at low temperatures (10–15°C).

Several studies have addressed the yeast cold stress adaptation topic. A transcriptomic analysis using QA23, a commercial *S. cerevisiae* wine-making strain, during low temperature industrial fermentations showed how the expression profiles at 25°C contrasted significantly with those at 13°C ^1^. In particular, the expression of genes associated with cell growth, cell cycle and maintenance was lower in the exponential phase at 13°C than at 25°C, and those genes activated in the exponential phase of growth at 13°C were essentially involved in environmental stress response ^2^.

Physiological and enological studies have suggested the potential benefits of the use of *S. kudriavzevii* in low temperature wine fermentations, and its cryotolerant nature ^3^. In a study comparing transcriptomes of *S. kudriavzevii* and *S. cerevisiae,* both species showed up-regulation of genes related to translational machinery, although *S. kudriavzevii* presented an enhanced response compared to *S. cerevisiae* ^4^. Tronchoni *et al.* ^4^ postulated that this response could be the result of alterations in the stability of a functional RNA conformation related to a competing structure. This suggests adaptation to cold shock in *S. kudriavzevii* due to higher ribosome availability and enhanced translation efficiency.

In cold European regions *S. kudriavzevii x S. cerevisiae* yeast hybrids are frequently used to produce wines. The enological characterization of several of those *S. cerevisiae* x *S. kudriavzevii* hybrids have suggested that the *S. cerevisiae* genome contributes to ethanol tolerance and elevated fermentative capacity ^5, 6^, whereas the *S. kudriavzevii* genome provides adaptation to low temperatures ^7, 8^. *S. kudriavzevii* produce higher amounts of glycerol during wine fermentations ^3, 5^, a characteristic that has been linked to cell survival in fermentations at low temperatures ^9^ and has also been found to be involved in freeze-thawing stress resistance ^10^.

This work presents a transcriptomic study of *S. cerevisiae* x *S. kudriavzevii* hybrids, aimed at deciphering the molecular adaptation of these strains to low temperatures. To perform this study, we used two wine yeast strains of *S. cerevisiae* x *S. kudriavzevii* hybrids that are commercialized in the market as cryophilic strains, Lalvin W27 and VIN7. These strains were selected according to previously published data ^6, 11^ and showed differences in genomic parental allele composition. Although both of them are allotriploid hybrids, the chromosomes inherited from each parent are different. W27 has lost part of chromosome IV, IX and XV of the *S. kudriavzevii* genome ^6^, whilst VIN7 has lost chromosomes III and parts of chromosomes IV and VII ^11, 12^.

## Methods

### Yeast strains and media

The yeasts strains included in this study were the strain Lalvin T73 ( *S. cerevisiae*) and the strain IFO1802 ( *S. kudriavzevii*), used as the parental species, and two *S. cerevisiae* x *S. kudriavzevii* hybrids, VIN7 and Lalvin W27, isolated from wine in South Africa and Switzerland, respectively. The yeast was grown and maintained in GPY medium (2% glucose, 0.5% peptone, 0.5% yeast extract) and plates (with 2% agar). Wine fermentations were performed in grape juice from the Tempranillo variety at 28°C or 12°C in vessels with 0.45 L of Tempranillo grape must.

### Total RNA extraction and cDNA labelling

Each yeast strain had two independent fermentations and three samples were taken from each independent fermentation. Cells from each yeast strain fermentation were pelleted by centrifugation (4000 rpm/min, 5 min), at 12°C and 28°C. Cells were collected at the beginning of the exponential phase by taking samples two generations after inoculation. The RNA extraction protocol was based on subsequent treatments with phenol-tris, phenol-chloroform (5:1) and chloroform-isoamyl alcohol (24:1), and an ethanol precipitation with sodium acetate ^13^. RNA concentrations and purity were determined using a Nanodrop spectrophotometer ND-1000 (Nanodrop Technologies™, Wilmington, DE). RNA integrity was checked by electrophoresis in agarose gel (1%). 2–4 μg of total RNA from each sample was linearly amplified using the Low RNA Input Fluorescent Linear Amplification kit (Agilent Technologies™, Ca, USA). 2–3 µg of amplified cRNA was used as template for cDNA synthesis. cDNA was marked indirectly using the SuperScript™ Indirect cDNA Labeling System (Invitrogen™, San Diego, CA). Cy3 and Cy5 mono-reactive Dye (Amersham GE Healthcare™, Amersham UK) were used as the fluorophores and dye incorporation was monitored using a Nanodrop spectrophotometer.

### cDNA hybridization

A 200 to 300 pmol mixture of the labelled cDNA samples was concentrated (Concentrator Plus, Eppendorf™, Hamburg, Germany). Competitive hybridization was performed on a Yeast 6.4K Array with PCR-amplified ORFs of yeast S288c strain (Microarray Centre, UHN, Toronto, Ontario, Canada) in AHC hybridization chambers (ArrayIt Corporation, CA, USA) at 42°C overnight. Heterologous conditions as stated by Gamero *et al.* ^14^ were employed to assure the hybridization of the *S. kudriavzevii* genome. The pre-hybridization solution contained 3X SSC, 0.1% SDS and 0.1 mg/ml BSA; the hybridization solution contained 0.1% SDS, 0.1 mg/ml of salmon DNA and 5X SSC. Microarrays were manually washed with different solutions containing different SSC 20X and SDS 10% concentrations (Sol.1: 0.1% SDS-2X SSC; Sol.2: 0.1% SDS-0.1X SSC; Sol.3: 0.1 SSC; Sol4: 0.01X SSC). The signal intensities of Cy3 and Cy5 were acquired with an Axon GenePix 4100A scanner (Molecular Devices, CA, USA) using GenePix Pro v.6.1 software, at a resolution of 10 µm.

### Microarray data analysis

Microarray data were derived from three independent cDNA hybridization experiments. Background correction was performed with GenePix pro 6.0. Subsequent analyses were performed with the Acuity 4.0 software (Molecular Devices, CA, USA). Individual datasets were normalized to log _2_ ratio value of 1 and data were filtered to remove spots that were not flagged and manually processed for print tip effect corrections. Only spots data with a minimum of two replicates were considered. Replicates were combined and medians were calculated. The first cut-off was the selection of the genes presenting at least 2-fold log _2_ ratio values, according to the literature ^15– 17^. These genes underwent a “GO term” enrichment analysis using the GO Term Finder tool in the *Saccharomyces* Genome Database ( http://www.yeastgenome.org/). Regarding the statistics, False Discovery Rate (FDR) analysis and a significance level of 99% (p value < 0.01) were applied.

### Quantitative PCR

Expression of genes *NUG1, LSM8, PDC5, NSR1, GPD1* and *GUT2* was investigated by qPCR. Normalization of gene expression was carried out using *ACT1* and *RDN18-1* as controls since their expression remains constant along fermentation. Primers were designed using Primer-BLAST (NCBI) and *S. kudriavzevii* and *S. cerevisiae* gene sequences were deposited in databases ( www.ncbi.nlm.nih.gov; GSE90793). Forward and reverse oligonucleotides were synthetized to hybridize to the selected alleles of *S. kudriavzevii* or *S. cerevisiae* genes ( Table 1). PCR Mastercycler pro (Eppendorf, Germany) confirmed the allele primer specificity and their annealing temperature. cDNA synthesis and RNA extraction were carried out as previously explained. qPCR runs were done in triplicate in a LightCycler® 480 Real-Time PCR System (Roche, Switzerland) and analyzed using the software from the manufacturer (LightCycler® Software, version 4.0). The relative gene expression was quantified by comparison with *ACT1* and *RDN18-1* expression, after confirm comparable PCR efficiency.

**Table 1.** Primers used in qPCR for allele expression analysis.

PRIMER	Organism	Sequence
NSR1 Sc F1	*S. cerevisiae/S. kudriavzevii*	CAAGAAGGAAGTTAAGGCTTCCAA
NSR1 Sc R9	*S. cerevisiae*	GAAGATGAAGATTCAGATTCAGACTCA
NSR1 Sk R7	*S. kudriavzevii*	TCGGAGGAAGAAGAGGTGCTT
NUG1c_Forw	*S. cerevisiae*	CTTAGAGGAAAGAGAGCTTGCCA
NUG1c_Rev	*S. cerevisiae*	CCATTTTCATCGTCTTCTATCATGT
NUG1k_Forw	*S. kudriavzevii*	TTTGGAAGAAAGAGAGCTTGCC
NUG1k_Rev	*S. kudriavzevii*	CCTTGTCATCTTCATCCACAACA
PDC5c Forw	*S. cerevisiae*	CGCTCCTACAAGACCAAAAATATC
PDC5c Rev	*S. cerevisiae*	CATTGGAGTGTTAGCTGGAGTAGAC
PDC5k Forw	*S. kudriavzevii*	CGCTCTTACAAGACAAAGAACGTT
PDC5k Rev	*S. kudriavzevii*	TGAGGTGTTAGCTGGGGTAGCT
GPD1c Forw	*S. cerevisiae*	CAATTGAAAGGTCATGTTGATTCA
GPD1c Rev	*S. cerevisiae*	TCAGTGATGTAAGAGGATAGCAATTG
GPD1k Forw	*S. kudriavzevii*	GAAAGGCCACGTTAACCCTC
GPD1k Rev	*S. kudriavzevii*	GGATAGAGCACCACATTGGATG
GUT2c Forw	*S. cerevisiae*	GGGGACGCTGTACTGGATG
GUT2c Rev	*S. cerevisiae*	ATCAACACGTCGAATTGATGC
GUT2k Forw	*S. kudriavzevii*	GGATCCGTGTACTGGGCG
GUT2k Rev	*S. kudriavzevii*	CAGCACATCGAATTGGTGC
LSM8c Forw	*S. cerevisiae*	ATGTCAGCCACCTTGAAAGACTAC
LSM8c Rev	*S. cerevisiae*	CTTGCAGATGAATTCCTTGCTT
LSM8k Forw	*S. kudriavzevii*	ATGTCGCCAATACTAAAGGAGTACTT
LSM8k Rev	*S. kudriavzevii*	GAGCCTTGCAGATGAACTCTTTAT
18S-F	*S. cerevisiae/S. kudriavzevii*	TTGCGATAACGAACGAGACC
18S-R	*S. cerevisiae/S. kudriavzevii*	CATCGGCTTGAAACCGATAG
ACT1_F	*S. cerevisiae/S. kudriavzevii*	GAAATGCAAACCGCTGCTCA
ACT1_R	*S. cerevisiae/S. kudriavzevii*	TACCGGCAGATTCCAAACCC

### Paromomycin assays

Yeast cells grown overnight in GPY were diluted to 2 × 10 ^6^ cells/ml the next morning. They were then grown until the mid-log phase (approximately 1 × 10 ^7^ cells/ml), and 175 μl were inoculated on each GPY plate. A filter (1 cm diameter) with paromomycin (2 μg) was placed on the surface and plates were incubated at 12°C or 28°C until the lawn was formed. The assays were repeated twice.

## Results

### *S. cerevisiae* x *S. kudriavzevii* hybrid strain fermentation performance under low temperature conditions

We carried out micro-fermentations in vessels with 0.45 L of Tempranillo grape must, mimicking previous work conditions ^4^. The time needed for both hybrid strains to finish the fermentation at 28°C was similar, 5 and 6 days for Lalvin W27 and VIN7, respectively ( Table 2). In contrast, at low temperature (12°C) the fermentation performance was quite different. W27 behaved more similar to the *S. kudriavzevii* type strain (11 days), taking 14 days to finish fermentation. Hybrid strain VIN7 performance was more similar to the *S. cerevisiae* parental strain (21 days) and took 23 days. The differences in fermentation kinetics amongst these strains revealed how allelic differences can determine the behaviour of oenological traits of interest.

**Table 2.** Time needed for the yeast strains to finish fermentation in Tempranillo grape must at 12°C and 28°C.

		12°C	28°C
Yeast species	Yeast strains	Fermentation (days)	
*S. cerevisiae*	T73	21	6
*S. kudriavzevii*	IFO 1802	11	11
Sc x Sk	Lalvin W27	14	5
	VIN7	23	6

### Differential gene expression in *S. cerevisiae* x *S. kudriavzevii* hybrids at low temperature

To evaluate changes in the global expression of genes during acclimation to low temperature in the wine fermentation of natural must, sampling was done at the beginning of the exponential phase when cells are growing at a speed close to µ _max_, two generations after inoculation. RNA from the samples was hybridised against the S288c microarray to study transcriptomic changes. An average of 86% sequence similarity exists between species of *S. cerevisiae* and *S. kudriavzevii* ^4^, so we used heterologous hybridisation conditions for the microarrays. We observed that 95% of the total S288c gene spots were hybridised by *S. kudriavzevii* DNA ^4^. Gene expression of each strain at both temperatures was analysed. Genes that were differentially expressed at 12°C and 28°C at a level that was considered significant were analysed further using the SAM (Significance Analysis of Microarrays) test with an FDR below 5% ( Supplementary Table 1).

In *S. cerevisiae* x *S. kudriavzevii* hybrid strain VIN7 at low temperature, 18 genes were up-regulated, while 22 genes were down-regulated. For W27, 20 genes were up-regulated while 3 genes were down-regulated. GO analysis using the GO Term Finder on the *Saccharomyces* Genome Database was performed to evaluate the GO categories arising from the up- and down-regulated genes in both hybrid strains ( Supplementary Table 2). Analysing the up-regulated genes in both strains after applying the GO-module online tool ( http://www.lussiergroup.org/GO-Module) for false positives resulted mainly in GO terms related to “magnesium ion binding”, “thiamine pyrophosphate binding”, “branched-chain-2-oxoacid decarboxylase activity” and “pyruvate decarboxylase activity”. The last category appeared, because amongst the up-regulated genes we found the three pyruvate decarboxylases *PDC1, PDC5* and *PDC6*, together with *IDP2*. The electron transport category was also significantly up-regulated in W27, similar to what has been previously described in *S. cerevisiae* and *S. kudriavzevii* ^4^. Amongst other up-regulated GO-terms in W27 is also “amino acid metabolism” with a number of sub-categories, and in “heavy metal binding” in VIN7. These GO-terms were also previously observed in *S. kudriavzevii*. For the hybrid strain VIN7 the genes down-regulated at 12°C were related to rRNA synthesis, while for W27 no GO term categories were found.

Overall, the most remarkable group of regulated genes (either up or down-regulated) in the hybrid strains fell into the translation machinery efficiency category, as previously shown for *S. kudriavzevii* compared to *S. cerevisiae* at 12°C ^4^. When comparing the two strains that showed high fermentation performance at 12°C ( *S. kudriavzevii* IFO1802 and the hybrid W27) to the two strains with low fermentation performance at 12°C ( *S. cerevisiae* T73 and VIN7), the cold adapted strains overexpressed 13 genes, including *NUG1,* involved in rRNA export ^18^, and chaperones *DDR48* ^19^ and *SRP1* ^20^ that couple proteasomes to polypeptides emerging from the ribosome ( Supplementary Table 1).

### Relative contribution of *S. cerevisiae* and *S. kudriavzevii* alleles to the total expression of key genes in the hybrids

An open question regarding yeast hybrids is the relative contribution of the different parental alleles to the total expression of specific genes. To test the role of the different alleles ( *S. cerevisiae* or *S. kudriavzevii)* in the better adaptation to cold temperatures, we selected three differentially overexpressed genes in W27 in this work, *NUG1*, *PDC5* and *LSM8,* and also three *S. kudriavzevii* cold stress markers described in different studies, *NSR1*, *GPD1* and *GUT2* ^3, 4, 21^. We observed overexpression of the overlapping dubious ORF *YJR023C* for *LSM8* and assumed that this overexpression was effectively in *LSM8*. *NSR1*, *LSM8* and *NUG1* are related to translation machinery efficiency, whereas *GPD1* and *GUT2* are related to glycerol metabolism, involved in cold adaptation. *GPD1* and *GUT2* are also involved in NAD ^+^/NADH balance, with *PDC5*. In Table 3 we included the genomic configuration, obtained from previous work ^6, 11, 12^, for the selected genes in each of the two hybrid strains, showing that most genes have at least one copy of each parental allele with the exception of *S. kudriavzevii* allele losses for *NSR1* in VIN7 and for *GPD1* and *GUT2* in the W27 strain. The results do not suggest a general correlation between the relative contribution of *S. kudriavzevii* alleles to total gene expression of key genes and the cryotolerance shown by W27.

**Table 3.** Allele-specific gene expression of relevant genes in hybrid strains W27 and VIN7 in Tempranillo grape must fermentation. Sc: *S. cerevisiae*; Sk: *S. kudriavzevii*. Sc/Sk is the relative expression of Sc alleles divided by relative expression of Sk. nd: not determined; <dl: below detection limit. C: *S. cerevisiae* allele; K: *S. kudriavzevii* allele. All values were normalized relative to the W27 *LSM8 S. cerevisiae* allele. *: Data obtained from previous work ^6, 11, 12^.

Hybrid strain	Gene	Genomic Composition*	Relative expression of allele		Ratio (Sc/Sk)	Total gene transcription levels
			*S. cerevisiae*	*S. kudriavzevii*		
W27	*NUG1*	CKK	299.01±19.21	40.30±3.65	7.42	339.31±19.55
	*PDC5*	CK	380.20±53.86	41.19±4.48	9.23	421.39±54.04
	*LSM8*	CK	1.00±0.12	6.58±0.16	0.15	7.58±0.19
	*NSR1*	CK	1275.25±102.97	186.14±12.48	6.85	1461.39±103.73
	*GPD1*	CC	895.05±19.11	<dl	-	895.05±19.11
	*GUT2*	CC	nd	<dl	-	<dl
VIN7	*NUG1*	CCK	392.08±14.75	65.54±2.20	5.98	457.62±14.91
	*PDC5*	CCK	296.04±22.38	16.24±6.53	18.23	312.28±23.31
	*LSM8*	CCK	0.62±0.07	4.11±0.40	0.15	4.73±0.41
	*NSR1*	CCC	895.05±19.11	<dl	-	895.05±19.11
	*GPD1*	CCK	648.51±14.16	1850.50±57.72	0.35	2499.01±59.43
	*GUT2*	CCK	113.86±13.86	81.98±1.84	1.39	195.84±13.98

W27 has one copy of the *NSR1 S. cerevisiae* allele, whereas VIN7 has three copies, but the *S. cerevisiae* allele expression is higher in the W27 strain. Thus, despite the higher number of total *S. cerevisiae* allele copies in VIN7, relative gene expression of the *S. cerevisiae NSR1* allele is higher in W27, and also total relative expression is higher in W27. One explanation is that the *S. kudriavzevii* allele in W27 may promote expression of both alleles. *NUG1* is another example of gene that does not show correlation between the number of copies and the level of expression. This gene has two copies of the *S. kudriavzevii* allele in W27 and one in VIN7 but shows higher expression in VIN7 than in W27. No expression was found for *GUT2*, but *GPD1* expression was remarkably higher in the VIN7 strain mainly due to the high *S. kudriavzevii* allele contribution. There is also a negative correlation between the copy number of the *S. cerevisiae* allele for the PDC5 gene and gene expression. Despite the similar expression of the *S. cerevisiae* allele for the PDC5 gene between both strains, we observe that the Sc/Sk ratio is higher in VIN7 than W27, which suggests this gene may have impact on efficiency of cold vinifications. The Sc/Sk ratio is the relative expression of *S. cerevisiae* alleles divided by the relative expression of *S. kudriavzevii* alleles.

### Effect of the translation inhibitor paromomycin

The expression of genes related to translation efficiency in the hybrid strains prompted us to study this phenotype. The translation efficiency of the hybrid strains compared to the parental strains at low temperature was analysed by testing their sensitivity to paromomycin, a potent inhibitor of translation ^22^.

**Figure 1.**
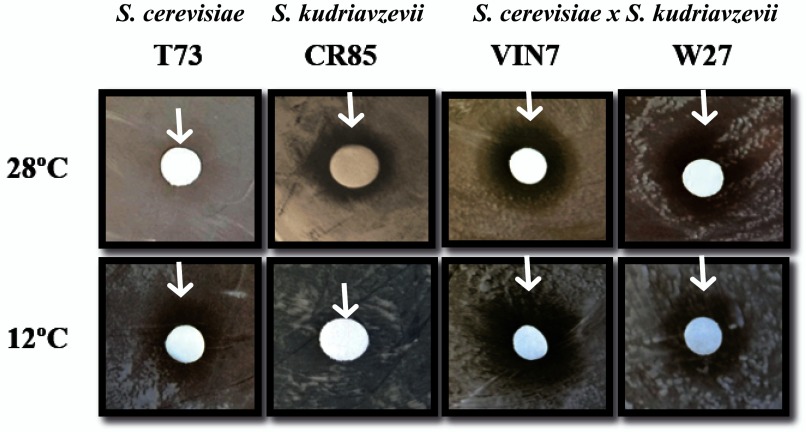
Evaluation of the translation efficiency of *S. cerevisiae*, *S. kudriavzevii* and *S. cerevisiae* x *S. kudriavzevii* hybrids. The inhibitory effect of the translation inhibitor paromomycin was evaluated by observing the halo diameter generated in the lawns of *S. cerevisiae* (T73), *S. kudriavzevii* (CR85) or *S. cerevisiae* x *S. kudriavzevii* hybrids (VIN7 and W27) growing in GPY plates at 28°C or 12°C. Representative experiments are shown, and the end of the inhibition halos are indicated with arrows.

As it can be seen in Figure 1, the *S. cerevisiae* strain shows a growth inhibition halo at 12°C, whereas no inhibition halo can be seen in the *S. kudriavzevii* strain at 12°C, confirming that the translation efficiency is not compromised for this yeast at low temperatures. *S. kudriavzevii* showed growth defects at 28°C, whereas *S. cerevisiae* remained unaffected. The hybrid strains, on the other hand, clearly show growth defects at both temperatures. This means that 28°C is not an optimal growth temperature for either of these hybrids. An important difference between the hybrid strains is that W27 shows a narrower halo at 12°C compared to 28°C while for the VIN7 strain the situation is the opposite, supporting the idea of W27 being more similar to *S. kudriavzevii* strains in low temperature conditions.

##  Discussion

In a previous paper, we analysed the transcriptomic behaviour of *S. cerevisiae* and *S. kudriavzevii* strains under low temperature fermentation conditions. Transcriptomic data showed the differences in gene expression between both species and also highlighted that the translation efficiency under low stress temperatures was higher in *S. kudriavzevii* than in *S. cerevisiae* ^4^. In this work, we wanted to further investigate the transcriptomics of *S. cerevisiae* x *S. kudriavzevii* hybrids at low temperature and compare the translation efficiency to previous data on *S. cerevisiae*. The results show that hybrids maintain the winemaking abilities classically attributed to *S. cerevisiae*, with increased expression of fermentation related genes. This explains their advantage in the winemaking environment over other natural species like *S. kudriavzevii,* which cannot compete with *S. cerevisiae* even in low temperature conditions ^7^. The hybrids also showed increased expression of genes related to cold adaptation, such as ribosome management genes ( *NUG1*, *SRP1*), and also displayed paromomycin resistance, which confirmed this adaptation. Resistance to paromomycin is the result of enhanced translation efficiency due to an increased number of ribosomes available to a new round of mRNA translation. We cannot discard that differences in paromomycin resistance are due to mutations in genes unrelated to translation, as it has been described before ^23– 26^. However, this possibility is unlikely since enhanced resistance phenotype at low temperatures has been observed in two different *S. kudriavzevii* strains ^4^ and, to less extent, in the two *S. cerevisiae* x *S. kudriavzevii* hybrids. In addition, our previous work have related paromomycin resistance with translation efficiency in these *S. kudriavzevii* strains by performing ^35^S-Methionine incorporation assay, showing that enhanced translation efficiency can be an adaptation to grow at low temperatures and alow adapted cell to shorten lag phase and resume earlier growth when cold is present ^4^. In total, these results suggest that hybrids maintain both fermentative and cold adaptation abilities attributed to each parental species. This highlights their industrially relevant characteristics.

In our attempt to determine the relative contribution of *S. cerevisiae* and *S. kudriavzevii* alleles to the total expression of genes in the hybrids, our results showed that allele copy number did not correlate with allele gene expression in the hybrid strains. Although the genomic contribution of *S. kudriavzevii* provides improved fermentation at colder temperatures, the reason cannot be explained solely by the presence of these allele copy numbers. It must be taken into account that the *S. kudriavzevii* genomic contribution in these hybrids is smaller than the *S. cerevisiae* genomic contribution, and probably under the control of *S. cerevisiae* genomic regulators. The equilibrium acquired between the genomes of *S. cerevisiae* and *S. kudriavzevii* in stable hybrid strains is the result of a complex process aiming to improve environmental adaptation, and cannot be explained only by the sum of both genomes.

## Data availability

The data referenced by this article are under copyright with the following copyright statement: Copyright: © 2017 Tronchoni J et al.

Data associated with the article are available under the terms of the Creative Commons Zero "No rights reserved" data waiver (CC0 1.0 Public domain dedication). 

The microarray data discussed in this publication can be found in the Gene Expression Omnibus database (NCBI), with accession number GSE90793.

## Supplementary material

**Supplementary Table 1. Transcriptomic dataset with genes that were differentially expressed at 12°C and 28°C genes in *S. cerevisiae* x *S. kudriavzevii* hybrids (VIN7 and W27) when compared to *S. cerevisiae* (T73) and *S. kudriavzevii* (CR85).** The genes that were considered significant were analysed further using the SAM (Significance Analysis of Microarrays) test with an FDR below 5%.

**Supplementary Table 2. GO analysis of the differentially expressed genes at different temperatures in *S. cerevisiae* x *S. kudriavzevii* hybrids (VIN7 and W27).** GO analysis using the GO Term Finder on the *Saccharomyces* Genome Database was performed to evaluate the GO categories arising from the up- and down-regulated genes in both hybrid strains. Analysis of the up-regulated genes in both strains was corrected for false positives after applying the GO-module online tool ( http://www.lussiergroup.org/GO-Module).
