# Supplementary material for: Transcriptomic analysis of Saccharomyces cerevisiae x Saccharomyces kudriavzevii hybrids during low temperature winemaking
Source: F1000Res. 2017 Sep 12;6:679. Originally published 2017 May 15. [Version 3] doi: 10.12688/f1000research.11550.3 (PMC5635440; doi:10.12688/f1000research.11550.3)
Supplement: Peer review history

# Peer review: Referee response for version 3

**DOI:** 10.5256/f1000research.13716.r25926

**Published:** 2017-10-06

**Authors:** Samuel Marguerat

**Affiliations:** 1 Institute of Clinical Sciences (ICS), Imperial College London, London, UK

The authors’ clarifications are useful and I now support the publication of their manuscript

I have read this submission. I believe that I have an appropriate level of expertise to confirm that it is of an acceptable scientific standard.

# Peer review: Referee response for version 2

**DOI:** 10.5256/f1000research.12757.r24099

**Published:** 2017-07-31

**Authors:** Samuel Marguerat

**Affiliations:** 1 Institute of Clinical Sciences (ICS), Imperial College London, London, UK

In this report Tronchoni and colleagues have analysed changes in mRNA expression in S. cerevisiae x S. kudriavzevii hybrids growing at different temperatures and compared them to strains from single species. They have also investigated the allelic expression of a series of relevant genes in the hybrids. Finally a growth inhibition experiment in presented in order to link their transcriptomic data to a temperature dependent modulation of the translation capacity of the hybrids. I list below a series of questions I have about this manuscript.

1. I couldn’t find any information about the division rate of the different cells at the time of collection. This is a crucial information as ribosome numbers change with division rate. I believe that including this information is important to support the authors’ interpretation of their data.
2. In general could the authors elaborate on what their model exactly is regarding the role of translational efficiency in their system please?
3. It is not clear to me whether the authors have checked the specificity of their species-specific qPCR primers on Sc or Sk strains. I may have missed it but otherwise it is an essential control that should be included.
4. I struggle with interpreting data from table 3. Is the efficiency of each primer pair comparable? Or is this taken case of by their design? Is the ratio Sc/Sk reflecting actual relative mRNA expression levels only or is it also reflecting differences in primer efficiencies? This should be clarified as a significant contribution of the latter to the data would impact on the authors’ interpretation of their results.
5. Figure 1 shows a representative experiment, it would be more rigorous to show a quantification of the size of the inhibition zone in each repeats so the reader can get an idea of how quantitative and reproducible this observation is. I am not familiar with this assay, but wouldn't it be possible to perform a dose response of the inhibition? Or maybe rather use a conventional drop test? This would greatly improve the data and help the reader understanding better this phenomenon.

I have read this submission. I believe that I have an appropriate level of expertise to confirm that it is of an acceptable scientific standard, however I have significant reservations, as outlined above.

# Response

**Published:** 2017-09-11

**Authors:** Roberto Pérez-Torrado

**Affiliations:** Institute of Agrochemistry and Food Technology (IATA), Spain

1. As the referee pointed out, growth rate affects ribosomes efficiency among many other things. Thus, we decide to sample at comparable situations. For this we sample not at the same time point but when cells were actively growing close to the maximal growth rate (a comment has been included in the results section), at the beginning of the exponential phase. Thus, we compare cells in a similar physiological and transcriptional situation.
2. We consider that cells with enhanced translation efficiency can be more adapted to low temperatures to better cope with cold and better resume high growth. A comment was included in the discussion section.
3. Yes, we did that control. Sentence was modified to clarify.
4. Yes, primers efficiencies were comparable. Sentence was modified to clarify.
5. We considered the reviewer suggestion. However, the halo zone are rather irregular with fuzzy borders, which complicates precise quantification. Thus, we decided to maintain the visual interpretation despite the loss of data clarity.

We appreciate all the reviewer suggestion that significantly enhanced the manuscript.

# Peer review: Referee response for version 2

**DOI:** 10.5256/f1000research.12757.r23334

**Published:** 2017-06-08

**Authors:** Matthias Sipiczki

**Affiliations:** 1 Department of Genetics and Applied Microbiology, University of Debrecen, Debrecen, Hungary

I have read this submission. I believe that I have an appropriate level of expertise to confirm that it is of an acceptable scientific standard.

# Peer review: Referee response for version 1

**DOI:** 10.5256/f1000research.12476.r22727

**Published:** 2017-05-30

**Authors:** Matthias Sipiczki

**Affiliations:** 1 Department of Genetics and Applied Microbiology, University of Debrecen, Debrecen, Hungary

This well-structured and nicely written paper demonstrates that the combinations of certain industrially relevant phenotypic properties of S. cerevisiae and S. kudriavzevii in strains of chimerical genomes comprised of different  proportions of their gene pools  (referred to as hybrids by the authors) cannot be simply attributed to differential expression of orthologous gene pairs assumed to be involved in the determination of those traits. The transcriptomic experiments were well designed and performed, and correctly interpreted.  As genes involved in the general translational machinery were identified among those overexpressed in strains with higher fermentation efficiency at low temperature, the authors compared the sensitivity of the strains to paromomycin, an agent that inhibits translation by binding to rRNA. The S. kudriavzevii laboratory strain (the parents of the chimerical strains are unknown) and the chimerical strain more active at the lower temperature were more resistant to the inhibitor. This finding was interpreted as indicating that their cells had higher numbers of ribosomes (to be inhibited by the drug) because of more active translation. This explanation is quite reasonable, but it should be mentioned in the Discussion that the increased resistance might also be due to mutations that make the RNA molecules of S. kudriavzevii less sensitive to the drug or impair its uptake at lower temperatures.  Numerous paromomycin-resistant Saccharomyces mutants have been described in the literature ^1^^-^^4^. In Kluyveromyces lactis, a membrane transporter specific for paromomycin was found ^5^. In Leishmania, the major mechanism of paromomycin resistance was found to be due to decreased drug uptake ^6^^,^^7^. The possibility that cold-sensitive mutations can also be involved in the different responses to paromomycin should be taken into consideration (and then refuted if the authors have good counterarguments) because all strains show the same growth intensity outside the inhibition zones at 12 °C in Fig. 1.

I have read this submission. I believe that I have an appropriate level of expertise to confirm that it is of an acceptable scientific standard, however I have significant reservations, as outlined above.

## References

[1] LiMTzagoloffAUnderbrink-LyonKMartinNC: Identification of the paromomycin-resistance mutation in the 15 S rRNA gene of yeast mitochondria. *J Biol Chem*.1982;257(10) :5921-8 6279619

[2] ColbyGWuMTzagoloffA: MTO1 codes for a mitochondrial protein required for respiration in paromomycin-resistant mutants of Saccharomyces cerevisiae. *J Biol Chem*.1998;273(43) :27945-52 977440810.1074/jbc.273.43.27945

[3] ZabetakisD: Inheritance of suppressors of the drug sensitivity of a NSR1 deleted yeast strain. *Yeast*.2000;16(12) : 10.1002/1097-0061(20000915)16:12<1147::AID-YEA610>3.0.CO;2-M 1147-59 10.1002/1097-0061(20000915)16:12<1147::AID-YEA610>3.0.CO;2-M 10953086

[4] SynetosDFrantziouCPAlksneLE: Mutations in yeast ribosomal proteins S28 and S4 affect the accuracy of translation and alter the sensitivity of the ribosomes to paromomycin. *Biochim Biophys Acta*.1996;1309(1-2) :156-66 895019010.1016/s0167-4781(96)00128-5

[5] ChenXJBauerBEKuchlerKClark-WalkerGD: Positive and negative control of multidrug resistance by the Sit4 protein phosphatase in Kluyveromyces lactis. *J Biol Chem*.2000;275(20) :14865-72 1080973010.1074/jbc.275.20.14865

[6] MaaroufMAdelineMTSolignacMVautrinDRobert-GeroM: Development and characterization of paromomycin-resistant Leishmania donovani promastigotes. *Parasite*.1998;5(2) : 10.1051/parasite/1998052167 167-73 10.1051/parasite/1998052167 9754313

[7] JhingranAChawlaBSaxenaSBarrettMPMadhubalaR: Paromomycin: uptake and resistance in Leishmania donovani. *Mol Biochem Parasitol*.2009;164(2) : 10.1016/j.molbiopara.2008.12.007 111-7 10.1016/j.molbiopara.2008.12.007 19146886PMC3039421

# Response

**Published:** 2017-05-30

**Authors:** Roberto Pérez-Torrado

**Affiliations:** Institute of Agrochemistry and Food Technology (IATA), Spain

We appreciate the reviewer comment and consider that it can significantly improve the paper. We have included this paragraph in the discussion to address the point raised by the reviewer:

 "We cannot discard that differences in paromomycin resistance are due to mutations in genes unrelated to translation as has been described before ^23-26^. However, this possibility is unlikely since enhanced resistance phenotype at low temperatures has been observed in two different *S. kudriavzevii* strains ^4^ and, less accused, in the two *S. cerevisiae* x *S. kudriavzevii* hybrids. In addition, our previous work have related paromomycin resistance with translation efficiency in these *S. kudriavzevii* strains by performing ^35^S-Methionine incorporation assay ^4^"

 Also, references suggested by the reviewer were incorporated.
